# Novel clinical risk scoring model for predicting mortality in patients with necrotizing fasciitis: The MNF scoring system

**DOI:** 10.1097/MD.0000000000028219

**Published:** 2021-12-23

**Authors:** Patcharin Khamnuan, Nipaporn Chuayunan, Acharaporn Duangjai, Surasak Saokaew, Natthaya Chaomuang, Pochamana Phisalprapa

**Affiliations:** a Department of Nursing, Phayao Hospital, Phayao, Thailand; b UNIt of Excellence on Clinical Outcomes Research and IntegratioN (UNICORN), School of Pharmaceutical Sciences, University of Phayao, Phayao, Thailand; c Center of Health Outcomes Research and Therapeutic Safety (Cohorts), School of Pharmaceutical Sciences, University of Phayao, Phayao, Thailand; d Department of Physiology, School of Medical Sciences, University of Phayao, Phayao, Thailand; e Division of Pharmacy Practice, Department of Pharmaceutical Care, School of Pharmaceutical Sciences, University of Phayao, Phayao, Thailand; f Unit of Excellence on Herbal Medicine, School of Pharmaceutical Sciences, University of Phayao, Phayao, Thailand; g Biofunctional Molecule Exploratory Research Group, Biomedicine Research Advancement Centre, School of Pharmacy, Monash University Malaysia, Bandar Sunway, Selangor Darul Ehsan, Malaysia; h Novel Bacteria and Drug Discovery Research Group, Microbiome and Bioresource Research Strength, Jeffrey Cheah School of Medicine and Health Sciences, Monash University Malaysia, Bandar Sunway, Selangor Darul Ehsan, Malaysia; i Division of Ambulatory Medicine, Department of Medicine, Faculty of Medicine Siriraj Hospital, Mahidol University, Bangkok, Thailand.

**Keywords:** MNF scoring system, necrotizing fasciitis, novel clinical risk scoring model, predicting mortality

## Abstract

Necrotizing fasciitis (NF) is a life-threatening soft tissue infection that rapidly progresses and requires urgent surgery and medical therapy. If treatment is delayed, the likelihood of an unfavorable outcome, including death, is significantly increased. The goal of this study was to develop and validate a novel scoring model for predicting mortality in patients with NF. The proposed system is hereafter referred to as the Mortality in Necrotizing Fasciitis (MNF) scoring system. A total of 1503 patients with NF were recruited from 3 provincial hospitals in Thailand during January 2009 to December 2012. Patients were randomly allocated into either the derivation cohort (n = 1192) or the validation cohort (n = 311). Clinical risk factors used to develop the MNF scoring system were determined by logistic regression. Regression coefficients were transformed into item scores, the sum of which reflected the total MNF score. The following 6 clinical predictors were included: female gender; age > 60 years; white blood cell (WBC) ≤5000/mm^3^; WBC ≥ 35,000/mm^3^; creatinine ≥ 1.6 mg/dL, and pulse rate > 130/min. Area under the receiver operating characteristic curve (AuROC) analysis showed the MNF scoring system to have moderate power for predicting mortality in patients with NF (AuROC: 76.18%) with good calibration (Hosmer-Lemeshow χ^2^: 1.01; *P* = .798). The positive likelihood ratios of mortality in patients with low-risk scores (≤2.5) and high-risk scores (≥7) were 11.30 (95% confidence interval [CI]: 6.16–20.71) and 14.71 (95%CI: 7.39–29.28), sequentially. When used to the validation cohort, the MNF scoring system presented good performance with an AuROC of 74.25%. The proposed MNF scoring system, which includes 6 commonly available and easy-to-use parameters, was shown to be an effective tool for predicting mortality in patients with NF. This validated instrument will help clinicians identify at-risk patients so that early investigations and interventions can be performed that will reduce the mortality rate among patients with NF.

## Abstract 2

Supplemental Digital Content is available in the text

## 1 Introduction

Necrotizing fasciitis (NF) is a life-threatening bacterial infection of the deep soft tissues that rapidly progressive and potentially fatal. Accurate diagnosis and treatment must require immediate surgical intervention and antimicrobial medication for reducing the NF mortality rate. If treatment is delayed, the likelihood of an unfavorable outcome, including death, is significantly increased.^[1,2]^

The number of cases reported for necrotizing fasciitis is 0.3 to 15 cases/100,000 population in nationwide study.^[2,3]^ The reported mortality rates as high as 15% to 36% and if untreated, reach 100%.^[4–7]^ In Thailand reported an NF incidence of 7.45 cases/100,000 population, and the fatality rate ranged 5.9% to 22.1%.^[8,9]^ Then, accurately assessing the severity of disease to predict the hospitalization and mortality in patients with NF is crucial.^[10]^

Accurate determination of the independent risk factors for mortality in patients with NF will help clinicians identify at-risk patients so that early investigations and interventions can be performed to improve outcomes and reduce mortality. The prognostic tools have been available to assess the severity of patients with NF on admission. The Acute Physiology and Chronic Health Evaluation II (APACHE II) and Simplified Acute Physiology Score II (SAPS II) scores are generated using the worst physiological characteristics obtained within the first 24 hours of intensive care unit admission (ICU).^[11,12]^ As a reported by a previous research, the APACHE II score was the most commonly utilized severity score for NF, while the Sequential Organ Failure Assessment (SOFA) and SAPS systems were less frequently used to forecast fatality in NF patients.^[10]^ Although theses prognostic tools for NF have been available, there is no epidemiology-based prognostic tool for NF in Thailand.

The purpose of this study was to develop and validate a novel, reliable, and easy-to-use scoring model for forecasting fatality in patients with NF based on the epidemiology of the disease in Thailand. The proposed system is hereafter referred to as the Mortality in Necrotizing Fasciitis (MNF) scoring system that will have boarder utility.

## 2 Methods

### 2.1 Study design and study size

The Transparent Reporting of a Multivariable Prediction Model for Individual Prognosis Or Diagnosis (TRIPOD) Statement was implemented in the development and validation of this retrospective study.^[13]^ The sample size was calculated using 10 outcome events per predictor variable (the EPV method).^[14]^ Based on the other scoring systems,^[15]^ which collectively draw upon 15 variables, a total of 150 patients were needed.

### 2.2 Setting and study population

Patients with confirmed NF who were admitted to three general hospitals in Northern Thailand. Those hospitals were Chiangrai Prachanukroh Hospital (a 600-bed tertiary care center), Kamphaeng Phet Hospital (a 330-bed secondary care center), and Phayao Hospital (a 400-bed secondary care center) during January 2009 to December 2012.

General hospital or provincial hospital in Thailand was defined as the hospitals which served as tertiary referral hospitals located in large provincial cities in 76 provinces across the country. General hospitals provide secondary to tertiary care and are the referral center within the province. At the provincial level, there is a general hospital covering a population of approximately 600,000. The size of general hospitals has large capacities with 150 to 500 beds. Some general hospitals have been upgraded to regional hospitals with 400 to 1000 beds and act as referral centres in particular regions. The largest regional hospitals have more than 1000 beds.^[16]^

NF was defined as widespread necrosis affecting at least involvement of the epidermis, dermis, subcutaneous tissue, fascia, and muscle. The term of mortality was defined as death within 28 days following surgery or death at the time of admission.^[17]^ Eligible subjects were patients diagnosed with NF. Diagnosis was made by surgeons who strictly observed the guidelines for skin and soft tissue infections published by the Infectious Diseases Society of America.^[1]^ A total of 1503 patients with NF were enrolled. Patients who had been definitively diagnosed with severe cellulitis were excluded.

All of the patients were assessed by emergency physicians and provided broad-spectrum antibiotics instantly. After investigation and evaluation, patients received appropriate emergency surgical treatment. Patient profiles were collected from inpatient charts. Using random sampling by computer generation (4:1),^[18]^ study patients were divided into either the derivation cohort (n = 1192) or the validation cohort (n = 311).

### 2.3 Model development

Variables found to be significantly associated with mortality in univariable logistic regression analysis were included in a multivariable logistic regression model to identify variables independently associated with mortality in NF. The coefficients of the variables obtained from multivariable analysis were weighted and then classified by the scores. The modification was accomplished by dividing each regression coefficient by the model's lowest coefficient and rounding the result to the nearest integer.^[19,20]^ A receiver operating characteristic (ROC) curve was constructed and a Hosmer-Lemeshow chi-square goodness of fit test was performed to measure the discriminative potential of the derived prediction score.^[21]^ According to their severity, cut-off scores were determined to put NF into 3 severity groups: low, moderate, and high-risk of mortality. The following parameters were calculated: sensitivity, specificity, positive predictive value (PPV), negative predictive value (NVP), positive likelihood ratio (LR+), and negative likelihood ratio (LR−).^[22,23]^

### 2.4 Model validation

The performance and accuracy of the score were examined by creating ROC curves in the validation cohort (n = 311). Prognostic performances of the score were compared between the derivation cohort and the validation cohort.

### 2.5 Statistical analysis

Exact probability tests and *t* tests were used to compare the baseline features of the development and validation data. In both sets of patients, severity ratings were assigned. The areas under the receiver operating curves (AuROC) were used to calculate the score's performance in the development and validation data. The probability curves for each of the severity levels demonstrated the score's discriminative potential. The results of that analysis are presented as adjusted odds ratio and 95% confidence interval (CI). All *P*-values were two-tailed, and a *P*-value of <.05 was considered statistically significant.

## 3 Results

### 3.1 Baseline characteristics

A total of 1503 patients with NF were enrolled. Their ages ranged from 2 to 95 years; 43.71% were female; 85.29% had a BMI ≥ 18.50; and 25.70% had diabetes mellitus (DM). A swelling wound was present in 82.48% of patients, and the most common wound site was the lower limbs (77.04%). The baseline characteristics of all patients are detailed in Table 1.

**Table 1:** Clinical and demographic characteristics of patients with necrotizing fasciitis.

Characteristics	All patients (N = 1503)	Derivation cohort (n = 1192)	Validation cohort (n = 311)
Gender			
Male	846 (56.29%)	685 (57.47%)	161 (51.76%)
Female	657 (43.71%)	507 (42.53%)	150 (48.24%)
Age (years)			
<60	691 (46.25%)	555 (46.91%)	136 (43.73%)
≥60	803 (53.75%)	628 (53.09%)	175 (56.27%)
Body mass index (kg/m^2^)			
≤18.50	197 (14.71%)	156 (14.83%)	41 (14.29%)
18.51–22.99	575 (42.94%)	443 (42.11%)	132 (45.99%)
≥23.00	567 (42.35%)	453 (43.06%)	114 (39.72%)
Education			
No education	652 (43.35%)	524 (43.92%)	128 (41.16%)
Primary education	763 (50.73%)	598 (50.13%)	165 (53.05%)
Secondary education	62 (4.12%)	48 (4.02%)	14 (4.50%)
Bachelor's degree or higher	26 (1.80%)	22 (1.93%)	4 (1.29%)
Occupation			
Older adult living at home	699 (46.41%)	555 (46.52%)	144 (46.01%)
Farmer/laborer	733 (48.67%)	574 (48.11%)	159 (50.80%)
Official	74 (4.91%)	64 (5.36%)	10 (3.19%)
Underlying morbidity			
Diabetes mellitus	387 (25.70%)	305 (25.54%)	82 (26.28%)
Heart disease	96 (6.38%)	74 (6.21%)	22 (7.05%)
Renal disease	45 (2.99%)	39 (3.27%)	6 (1.92%)
Cirrhosis	61 (4.05%)	55 (4.61%)	6 (1.92%)
Hypertension	538 (35.70%)	434 (36.35%)	104 (33.23%)
Gout	147 (9.75%)	116 (9.72%)	31 (9.90%)
Chronic alcoholism	232 (15.39%)	192 (16.08%)	40 (12.78%)
Wound characteristics			
Swelling	1,243 (82.48%)	990 (82.91%)	253 (80.83%)
Erythema	774 (51.36%)	614 (51.42%)	160 (51.12%)
Bleb	651 (43.20%)	515 (43.13%)	136 (43.45%)
Skin necrosis	403 (26.74%)	329 (27.55%)	74 (23.64%)
Gangrene	37 (2.46%)	28 (2.35%)	9 (2.88%)
Severe pain	1,316 (87.38%)	1,045 (87.52%)	271 (86.86%)
Site of wound			
Head and neck	8 (0.53%)	8 (0.67%)	0 (0.0%)
Trunk	28 (1.86%)	26 (2.18%)	2 (0.64%)
Upper limb	276 (18.31%)	219 (18.34%)	57 (18.21%)
Lower limb	1,161 (77.04%)	913 (76.47%)	248 (79.23%)
Fournier's gangrene	29 (1.92%)	23 (1.93%)	6 (1.92%)
Multiple sites	5 (0.33%)	5 (0.42%)	0 (0.0%)
Hospital			
Chiang Rai	817 (54.21%)	649 (54.36%)	168 (53.67%)
Kamphaeng Phet	557 (36.96%)	429 (35.93%)	128 (40.89%)
Phayao	133 (8.83%)	116 (9.72%)	17 (5.43%)
Laboratory on admission			
White blood cell count (/mm^3^)	16,903.28 ± 236.53	16,783.53 ± 253.48	17,357.72 ± 601.03
Polymorphonuclear cell or neutrophil (%)	82.05 ± 0.32	81.96 ± 0.37	82.41 ± 0.61
Creatinine (mg/dL)	1.92 ± 0.03	1.95 ± 0.04	1.8 ± 0.08
Bicarbonate (mmol/L)	21.68 ± 0.20	21.51 ± 0.22	22.27 ± 0.41
Total protein (g/dL)	6.33 ± 0.04	6.36 ± 0.05	6.25 ± 0.09
Laboratory 48–72 h			
White blood cell count (/mm^3^)	14,515.36 ± 356.47	14,132.02 ± 367.71	16,256.68 ± 1,041.54
Polymorphonuclear cell or neutrophil (%)	78.71 ± 0.64	78.44 ± 0.69	79.95 ± 1.73
Creatinine (mg/dL)	2.25 ± 0.08	2.26 ± 0.09	2.23 ± 0.22
Bicarbonate (mmol/L)	22.51 ± 1.33	23.09 ± 1.67	20.47 ± 1.24
Total protein (g/dL)	5.63 ± 0.11	5.62 ± 0.13	5.67 ± 0.17
Vital signs on admission			
Body temperature (°C)	37.31 ± 0.02	37.30 ± 0.02	37.34 ± 0.04
Pulse rate (/min)	91.40 ± 0.41	91.62 ± 0.46	90.55 ± 0.92
Respiration rate (/min)	20.15 ± 0.09	20.12 ± 0.10	20.30 ± 0.22
Systolic blood pressure (mm Hg)	117.16 ± 0.62	117.36 ± 0.70	116.41 ± 1.38
Diastolic blood pressure (mm Hg)	70.21 ± 0.38	70.22 ± 0.42	70.20 ± 0.84
Vital signs 48–72 h			
Body temperature (°C)	37.26 ± 0.02	37.26 ± 0.02	37.28 ± 0.04
Pulse rate (/min)	87.74 ± 0.38	87.42 ± 0.42	88.94 ± 0.87
Respiratory rate (/min)	19.46 ± 0.11	19.47 ± 0.12	19.43 ± 0.26
Systolic blood pressure (mm Hg)	120.69 ± 0.47	120.81 ± 0.55	120.26 ± 0.95
Diastolic blood pressure (mm Hg)	73.10 ± 0.30	72.95 ± 0.34	73.64 ± 0.65
Treatment and outcomes			
Incision and drainage	45 (2.99%)	38 (3.18%)	7 (2.24%)
Debridement	962 (63.84%)	757 (63.40%)	205 (65.50%)
Fasciotomy	654 (43.43%)	517 (43.34%)	137 (43.77%)
Amputation	127 (8.43%)	99 (8.29%)	28 (8.95%)
Severe sepsis	239 (16.37%)	186 (16.12%)	53 (17.32%)
Length of hospital stay (days)	11.29 ± 0.32	11.43 ± 0.33	10.74 ± 0.90

Data presented as number and percentage or mean ± standard deviation.

### 3.2 Indicator parameters of mortality in patients with NF

The findings of a univariable analysis of the derivation cohort are presented in Table 2. Sixteen risk predictors were found to be significantly associated with mortality in NF, including female gender, age, education level, heart disease, hypertension, erythema wound, bleb wound, WBC count, polymorphonuclear cell or neutrophil, creatinine level, bicarbonate level, pulse rate, systolic blood pressure, diastolic blood pressure, severe sepsis, and length of hospital stay. The overall fatality rate was 19.3% (290 of 1503 patients).

**Table 2:** Univariable analysis for risk factors significantly associated with mortality in patients with necrotizing fasciitis in the derivation cohort.

	Derivation cohort (n = 1192)		
Factors	Odds ratio	95% CI of odds ratio	*P*
Female gender	1.53	1.13–2.07	***.0036***
Age (per year)	2.15	1.57–2.97	***<.001***
Body mass index	1.34	0.96–1.89	.0720
Education	1.97	1.45–2.69	***<.001***
Occupation	1.63	1.20–2.21	***.001***
Underlying morbidity			
Diabetes mellitus	1.20	0.85–1.67	.2622
Heart disease	2.61	1.52–4.40	***<.001***
Renal disease	1.68	0.74–3.55	.1472
Cirrhosis	2.76	1.48–5.00	***.0003***
Hypertension	1.87	1.38–2.53	***<.001***
Gout	1.96	1.24–3.05	***.0016***
Chronic alcoholism	1.17	0.77–1.81	.4406
Wound appearance			
Swelling	1.32	0.90–1.92	.1258
Erythema	1.96	1.44–2.67	***<.001***
Bleb	2.15	1.59–2.92	***<.001***
Skin necrosis	1.05	0.75–1.46	.7370
Gangrene	1.09	0.40–3.72	.8556
Severe pain	2.13	1.23–3.91	***.0051***
Site of wound			
Head and neck	0	0–2.01	.1666
Trunk	2.70	1.08–6.42	***.0117***
Upper limb	1.03	0.70–1.55	.8438
Lower limb	1.19	0.84–1.67	.2937
Fournier's gangrene	6.38	0.72–76.68	***.0203***
Multiple sites	1.49	0.47–4.03	.3968
Laboratory on admission			
White blood cell count (/mm^3^)	4.55	2.82–7.28	***<.001***
Polymorphonuclear cell or neutrophil (%)	2.42	1.76–3.34	***<.001***
Creatinine (mg/dL)	4.85	3.44–6.89	***<.001***
Bicarbonate (mmol/L)	9.83	4.71–20.62	***<.001***
Total protein (g/dL)	12.30	2.48–117.93	***.0001***
Vital signs on admission			
Body temperature (°C)	1.41	1.03–1.93	***.0245***
Pulse rate (/min)	12.05	4.40–37.94	***<.001***
Systolic blood pressure (mm Hg)	5.54	3.62–8.43	***<.001***
Diastolic blood pressure (mm Hg)	4.70	2.84–7.73	***<.001***
Treatment and outcomes			
Incision and drainage	0.62	0.18–1.64	.3368
Debridement	0.77	0.57–1.05	.0899
Fasciotomy	1.04	0.77–1.40	.7815
Amputation	1.07	0.60–1.81	.7906
Severe sepsis	56.22	35.5–89.28	***<.001***
Length of hospital stay (days)	0.49	0.36–0.67	***<.001***

A *P*-value < .05 indicates statistical significance. CI = confidence interval.

### 3.3 Model development

The variables identified in univariable analysis were then entered into multivariable analysis to develop the scoring system for mortality in patients with NF. Using backward stepwise logistic regression, 6 variables remained statistically significant in the multivariable model. The score for predicting mortality in patients with NF is reflected by the summation of the point value from each of the following factors: female gender (yes = 1, no = 0); age > 60 (yes = 1, no = 0); WBC ≤ 5000/mm^3^ (yes = 3.5, no = 0); WBC ≥ 35,000/mm^3^ (yes = 2, no = 0); creatinine ≥ 1.6 mg/dL (yes = 2.5, no = 0); and, pulse rate > 130/min (yes = 4, no = 0). The point value for each factor was derived from the weighted coefficient, and then rounded to its nearest integer (Table 3).

**Table 3:** Multivariable analysis to identify independent predictors of mortality in patients with necrotizing fasciitis, and determination of the assigned score for each predictor.

Predictors	Coefficient	Adjusted OR	95% CI of adjusted OR	*P*	Assigned score
Female gender	0.613525	1.84	1.32–2.57	***<.001***	1
Age >60 years	0.557956	1.74	1.22–2.48	***.002***	1
WBC ≤5,000/mm^3^	2.046881	7.74	4.06–14.75	***<.001***	3.5
WBC ≥35,000/mm^3^	1.005165	2.73	1.27–5.85	***.010***	2
Creatinine ≥1.6 mg/dL	1.463608	4.32	3.02–3.02	***<.001***	2.5
Pulse rate > 130/min	2.243062	9.42	3.02–26.33	***<.001***	4

A *P*-value < .05 indicates statistical significance. CI = confidence interval, OR = odds ratio, WBC = white blood cell count.

**Figure 1:**
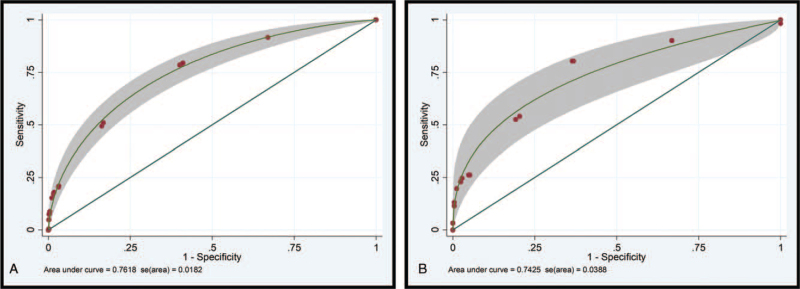
ROC curve of the scoring system in predicting mortality in patients with necrotizing fasciitis in (A) derivation cohort (n = 1192) and (B) validation cohort (n = 311). ROC = receiver operator characteristic.

The risk-scoring system was created by defining the cut-off based on the discrimination plot and the clinical predict parameter performance. Cut-off scores of 2.5 and 7 were classified patients into 3 severity groups. Patients with a total score of ≥7 were categorized into the high-risk group. Mortality among those patients was found to be predicted with high accuracy (35/45 cases; PPV: 77.78%). Patients with a total score of ≤2.5 were categorized into the low-risk group. A comparison between those who died and those who did not die in the low-risk group revealed that survival was correctly predicted in 92.23% of cases (570/618). The absence of mortality could be excluded with moderate accuracy (NPV: 31.53%). The corrected prediction of absence or presence of mortality was (570 + 35)/(618 + 45) = 91.25%, while the incorrect prediction rate was (48 + 10)/(618 + 45) = 8.74% (Table 4). Using this scoring system and 2 cut-off points, the score could discriminate between those with and without risk of mortality with satisfactory validity (AuROC 76.18%) (Fig. 1). The predictive model was also found to be well-calibrated (Hosmer-Lemeshow χ^2^ = 1.01; *P* = .799).^[24]^

**Table 4:** Risk of mortality in patients with necrotizing fasciitis compared among the low-, moderate-, and high-risk groups, and diagnostic performance and interpretation in the derivation cohort (n = 1192).

Derivation cohort	Low-risk (score ≤2.5)	Moderate-risk (score 3–6.5)	High-risk (score ≥7)	Total
Total	618	529	45	1192
Not deceased	570	383	10	963
Deceased	48	146	35	229
Diagnostic performance				
Sensitivity	59.19%		15.28%	
Specificity	79.03%		98.96%	
PPV	92.23%		77.78%	
NPV	31.53%		83.08%	
Likelihood ratio (+)	11.30 (95% CI: 6.16–20.71)		14.71 (95% CI: 7.39–29.28)	
Likelihood ratio (−)	0.43 (95% CI: 0.39–0.46)		0.85 (95% CI: 0.8–0.90)	

CI = confidence interval, NPV = negative predictive value, PPV = positive predictive value.

### 3.4 Model validation

The ROC curves for the development and validation cohorts showed similar results (AuROC 76.18% and 74.25%, respectively; Fig. 1). The high-risk group were accurately predicted in 80.00% of cases, and the presence of mortality was diagnosed with high accuracy (PPV: 80.00%). In the low-risk group, survival was correctly predicted in 92.94% (158/170) of cases, with the absence of mortality being predicted with moderate accuracy (NPV: 34.75%). The accurately prediction of absence or presence of mortality was (158 + 12)/(170 + 15) = 91.89%, while the incorrect prediction rate was (12 + 3)/(170 + 15) = 8.11% (Table 5).

**Table 5:** Risk of mortality in patients with necrotizing fasciitis compared among the low-, moderate-, and high-risk groups, and diagnostic performance and interpretation in the validation cohort (n = 311).

Validation cohort	Low-risk (score ≤2.5)	Moderate-risk (score 3–6.5)	High-risk (score ≥7)	Total
Total	170	126	15	311
Not deceased	158	89	3	250
Deceased	12	37	12	61
Diagnostic performance				
Sensitivity	63.20%		19.67%	
Specificity	80.33%		98.80%	
PPV	92.94%		80.00%	
NPV	34.75%		83.45%	
Likelihood ratio (+)	10.95 (95% CI: 3.63–32.99)		16.39 (95% CI: 4.77–56.29)	
Likelihood ratio (−)	0.39 (95% CI: 0.32–0.46)		0.81 (95% CI: 0.71–0.92)	

CI = confidence interval, NPV = negative predictive value, PPV = positive predictive value.

**Figure 2:**
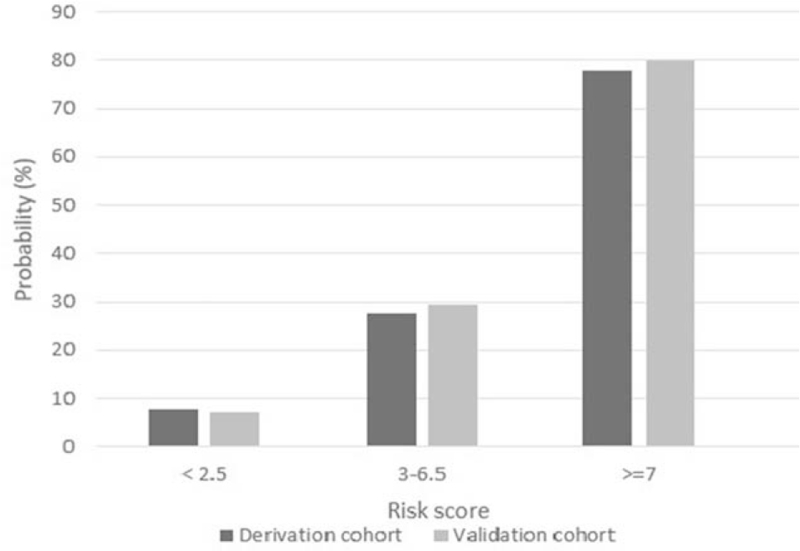
Probability of mortality in patients with necrotizing fasciitis, stratified by the risk score.

The probability of risk of mortality in patients with NF as a function of the risk score is illustrated in Figure 2. Patients were classified into 3 groups following cut-off points: low-risk (score ≤ 2.5); moderate-risk (score of 3–6.5); and high-risk (score ≥ 7).

## 4 Discussion

This study set forth to establish a clinical risk-scoring system to clinical prognostic mortality in patients with NF: The MNF scoring system. We established that this validated clinical risk scoring system can be used as a prognostic tool to identify patients with NF who need further management. The MNF score was able to identify high-risk patients with NF requiring early investigation and treatment. This is the first study to establish a scoring system to evaluate mortality risk among patients with NF in Thailand, which is a low-to-middle income Asian country.

Patients with NF were divided into the mortality and survival groups. The MNF score was shown to be an effective prognostic tool for predicting the risk of death in patients with NF, with the direct implication that these patients would receive expedited evaluation and care. The score draws upon predictors related to a patient's demography; clinical signs, symptoms, and appearance; vital signs; and, laboratory profiles. Using a scheme developed from 6 predictors (female gender, age > 60 years, WBC ≤ 5000/mm^3^, WBC ≥ 35,000/mm^3^, creatinine ≥ 1.6 mg/dL, and pulse rate > 130/min), patients with NF in this study were classified into 3 groups according to their likelihood of mortality. The MNF score was validated, and it demonstrated high discriminative power when applied to the validation cohort. The cut-off point in this study was based on evaluations of sensitivity, specificity, and positive and negative predictive values. The aim of using the MNF score to predict mortality in patients with NF is that it will alert emergency department clinicians of the need to provide rapid treatment. The cut-off point was determined to be an MNF score of ≥7. Under the MNF scoring system, patients scoring ≤2 are categorized into the low-risk group, which does not require emergency debridement for NF. Patients scoring 3 to 6.5 are categorized into the moderate-risk group, which requires further investigations according to each patient's underlying conditions and the judgment of the physician. Among patients with high mortality risk (those with an MNF score ≥7), the rapid administration of emergency operative debridement and broad-spectrum antibiotic therapy is recommended to reduce the risk of mortality.

The MNF score developed in this study differs from APACHE II scores.^[11,25]^ APACHE II employs a prognostic scoring system for critical care that includes oxygenation or PaO_2_; vital signs (temperature, heart rate, mean arterial pressure, and respiratory rate); and, laboratory metabolic parameters (serum sodium, potassium, creatinine, bicarbonate concentrations, WBC count, and hematocrit).^[11]^

The present study constructed a prognostic scheme based on 6 predictors (female gender, age > 60 years, WBC ≤ 5,000/mm^3^, WBC ≥ 35,000/mm^3^, creatinine level ≥ 1.6 mg/dL, and pulse rate > 130/min) that were identified in a multivariable analysis (Table 3). These predictors are similar to the risk factors for mortality identified by many other studies.^[1,5,15,26]^ Gender could predict mortality and affected the treatment outcomes. Being female demonstrated an increased risk for mortality, which is consistent with earlier research that found a significantly higher number of deceased females compared to males. A possible reason for this is that females have more subcutaneous fat than males, making them more susceptible to infection.

Concerning age, older adults are widely considered to have worse prognostic factors than their younger age group counterparts. Several previous studies reported advanced age to be an independent factor for mortality.^[15,26–28]^ The present study also found patients with NF aged >60 years to be at increased risk for mortality.

We also found a pulse rate > 130 beats/min to be independently associated with increased mortality risk. A previous study found that a high heart rate results from the septic shock that occurs in patients with NF with sepsis.^[29]^ Septic shock was reported to be a serious complication in patients with NF.^[30]^ Moreover, patients with a pulse rate over 130 beats per minute can be predicted to experience septic shock, which earlier studies was reported to be an important risk factor for organ failure and fatality.^[31–34]^

Sepsis was common cause of death worldwide, and WBC count was found to be associated with a greater risk of death in patients with NF.^[35]^ Previous studies reported that increased serum creatinine levels could be used to predict impaired renal function that was most likely associated with septic shock, and that high creatinine levels might indicate renal failure.^[15,26,27,36]^ We collected clinical data to examine whether sepsis could be predicted. In our study, patients were considered to have sepsis if their WBC count was ≤5,000 or ≥35,000/mm^3^. Our multivariable analysis revealed laboratory findings of a serum creatinine level ≥1.6 mg/dL, a WBC count of ≤5,000/mm^3^, and a WBC count of ≥35,000/mm^3^ all to be independent risk factors for death in patients with NF.

### 4.1 Strengths

The strengths of this study should be acknowledged. First, this study consisted of a large sample size of patients with NF to assess mortality outcome. We also included patients from 3 large hospitals in Thailand, which recommends that our findings may be applied to other parts of Thailand as well as other low- and middle-income Asian countries. The MNF scoring system will assist general hospitals in rural areas, such as provincial and community hospitals. Because it is a simple-to-use routine standard laboratory for forecasting and monitoring the risks of illness progression and mortality. Second, the MNF risk scoring system was developed in accordance with the stringent criteria set forth in the TRIPOD statement.^[13]^ Third, the developed scoring system includes only 6 variables, all of which are easy to obtain and input to obtain the total MNF score. These 6 predictors are easily obtained from a patient's demography (female gender; aged > 60 years), clinical characteristics (pulse rate > 130/min), and routine laboratory results (WBC ≤ 5,000/mm^3^; WBC ≥ 35,000/mm^3^; serum creatinine level). Fourth, the MNF scoring system was validated using different patient data sets. The MNF score showed good prediction capability with acceptable diagnostic performance in both the derivation and validation cohorts. Fifth and last, the MNF model is inexpensive since only 2 laboratory investigations are required (WBC count and serum creatinine).

### 4.2 Limitations

The fundamental limitation in this study is that it was conducted retrospectively, making it prone to missing or incomplete data. Further studies should be conducted to compare MNF score discrimination between survivors and non-survivors, and to confirm our findings. Despite these acknowledged limitations, the MNF scoring system can be used in routine health care services due to its low cost and user friendliness. The implication of the MNF score is that its application may lead to rapid identify of a risk of mortality in patients with NF. Via the use of this scoring system, disease progression can be retarded, potential complications of NF can be monitored, and the risk of death can be greatly reduced.

## 5 Conclusions

A simple scoring system for the prediction of mortality in patients with NF has been developed and validated. The proposed MNF scoring system, which includes 6 commonly available and easy-to-use parameters, was shown to be an effective tool for predicting mortality in patients with NF. This validated instrument will help clinicians identify at-risk patients so that early proper management can be performed that will reduce the fatality rate among patients with NF (Supplementary Table S1, http://links.lww.com/MD2/A759, Diagram, http://links.lww.com/MD2/A760).

## Acknowledgments

The authors gratefully acknowledge Chiangrai Prachanukroh Hospital, Kamphaeng Phet Hospital, and Phayao Hospital for their support of this study. We would also like to thank Mr. David Park and Kevin P. Jones (medical manuscript editors), Siriraj Medical Research (SiMR), Faculty of Medicine Siriraj Hospital, Mahidol University, Bangkok, Thailand for their critical review and revision of the manuscript.

## Author contributions

Study concept and design: PK, NaC, and SS; Data acquisition: PK, NiC, and SS; Statistical analysis and data interpretation: PK, NaC, and SS; Drafting of the manuscript: NaC, SS, and PP; and Critical revision of the manuscript: PK, NiC, AD, SS, NaC, and PP. All authors have approved the final submitted version of the manuscript, and all authors agree with the decision to submit this manuscript for journal publication.

**Conceptualization:** Patcharin Khamnuan, Surasak Saokaew, Natthaya Chaomuang.

**Data curation:** Patcharin Khamnuan, Nipaporn Chuayunan, Surasak Saokaew.

**Methodology:** Natthaya Chaomuang.

**Supervision:** Acharaporn Duangjai, Surasak Saokaew, Pochamana Phisalprapa.

**Writing – original draft:** Surasak Saokaew, Natthaya Chaomuang, Pochamana Phisalprapa.

**Writing – review & editing:** Patcharin Khamnuan, Surasak Saokaew, Natthaya Chaomuang, Pochamana Phisalprapa.
